# Benchmarking explanation methods for mental state decoding with deep learning models

**DOI:** 10.1016/j.neuroimage.2023.120109

**Published:** 2023-04-13

**Authors:** Armin W. Thomas, Christopher Ré, Russell A. Poldrack

**Affiliations:** a Stanford Data Science, Stanford University, 450 Serra Mall, 94305, Stanford, USA; b Dept. of Psychology, Stanford University, 450 Serra Mall, Stanford, 94305, USA; c Dept. of Computer Science, Stanford University, 450 Serra Mall, 94305, Stanford, USA

**Keywords:** Neuroimaging, Mental state decoding, Deep learning, Explainable AI, Benchmark

## Abstract

Deep learning (DL) models find increasing application in mental state decoding, where researchers seek to understand the mapping between mental states (e.g., experiencing anger or joy) and brain activity by identifying those spatial and temporal features of brain activity that allow to accurately identify (i.e., decode) these states. Once a DL model has been trained to accurately decode a set of mental states, neuroimaging researchers often make use of methods from explainable artificial intelligence research to understand the model’s learned mappings between mental states and brain activity. Here, we benchmark prominent explanation methods in a mental state decoding analysis of multiple functional Magnetic Resonance Imaging (fMRI) datasets. Our findings demonstrate a gradient between two key characteristics of an explanation in mental state decoding, namely, its faithfulness and its alignment with other empirical evidence on the mapping between brain activity and decoded mental state: explanation methods with high explanation faithfulness, which capture the model’s decision process well, generally provide explanations that align less well with other empirical evidence than the explanations of methods with less faithfulness. Based on our findings, we provide guidance for neuroimaging researchers on how to choose an explanation method to gain insight into the mental state decoding decisions of DL models.

Deep learning (DL) models have celebrated immense successes in many areas of research and industry (Goodfellow et al., 2016; LeCun et al., 2015). This success is often attributed to their unmatched ability to learn versatile representations of complex datasets, allowing them to associate a target signal with varying patterns in minimally-preprocessed (or raw) data. Due to their empirical success, neuroimaging researchers have started applying DL models to mental state decoding analyses (e.g., Mensch et al., 2021; Plis et al., 2014; Thomas et al., 2019; 2022a; VanRullen and Reddy, 2019; Wang et al., 2020; Zhang et al., 2021). In these analyses, researchers seek to understand how specific mental states (e.g., answering questions about a prose story or math problem) are represented in the brain by identifying brain regions (or networks of brain regions) whose activity patterns allow accurate identification (i.e., decoding) of these mental states (Haynes and Rees, 2006).

Once a DL model has been trained to accurately decode a set of mental states from brain activity, researchers often make use of explanation methods from explainable artificial intelligence research (XAI; Doshi-Velez and Kim, 2017; Samek et al., 2021) to gain insights into the models’ learned mappings between mental states and brain activity, seeking to overcome the black box nature of DL models (e.g., Dinsdale et al., 2021; Kohoutov et al., 2020; Oh et al., 2019; Thomas et al., 2019; Wang et al., 2020; Zhang et al., 2022). An explanation of a mental state decoding decision of a DL model thereby seeks to provide human-understandable insight into how the model maps the decoded mental state to the input brain activity. From the wealth of existing explanation methods (Gilpin et al., 2018; Linardatos et al., 2021), neuroimaging researchers most often utilize attribution (i.e., “heatmapping”) methods, which attribute a relevance to each feature value of an input for the resulting decoding decision of a DL model, resulting in a heatmap of relevance values (Samek et al., 2021). Accordingly, the resulting explanations are specific to the features of the input brain data and resulting mental state decoding decision of the DL model.

At a high level, prominent attribution methods in mental state decoding can be grouped into sensitivity analyses (e.g., Simonyan et al., 2014; Smilkov et al., 2017; Springenberg et al., 2015), reference-based attributions (e.g., Lundberg and Lee, 2017; Shrikumar et al., 2017; Sundararajan et al., 2017), and backward decompositions (e.g., Bach et al., 2015; Montavon et al., 2017). Sensitivity analyses, such as Gradient Analysis (Simonyan et al., 2014), attribute relevance to input features according to how sensitively a model’s behavior (i.e., decoding decision) responds to a feature’s value. Reference-based attributions, such as Integrated Gradients (Sundararajan et al., 2017), by contrast, attribute relevance to input features by comparing the model’s response to a given input to its response to a reference input (e.g., a neutral input). Backward decompositions, such as Layer-wise Relevance Propagation (LRP; Bach et al., 2015), on the other hand, attribute relevance to input features by decomposing the decoding decision of a DL model in a backward pass through the model into the contributions of lower-level model units to the decision, up to the input space, where a contribution for each input feature can be defined.

Given the wealth of existing attribution methods, neuroimaging researchers interested in explaining the mental state decoding decisions of DL models are faced with the task of choosing a method for their particular analysis and research question. Yet, in many cases, the explanations of different attribution methods are difficult to visually discern, making it challenging to compare and evaluate their quality. Even further, it is unclear whether related empirical findings from computer vision (CV; Adebayo et al., 2018; Kindermans et al., 2019; Samek et al., 2017) and natural language processing (NLP; Ding and Koehn, 2021; Jacovi and Goldberg, 2020; Jain and Wallace, 2019) regarding the relative performance of prominent attribution methods are generalizable to mental state decoding. There, researchers have often argued that reference-based attributions and backward decompositions are superior to sensitivity analyses because they capture the decision process of a DL model more faithfully. Yet, mental state decoding is distinct from most CV and NLP applications in that researchers seek to understand the association of input data (i.e., brain activity) and decoding targets (i.e., mental states), whereas CV and NLP are often solely concerned with predictive performances (Lipton and Steinhardt, 2018). To date, it is therefore unclear how prominent attribution methods compare in providing insights into the mappings between brain activity and mental states learned by DL models.

In the present work, we compare the explanation performance of prominent attribution methods in a mental state decoding analysis of three functional Magnetic Resonance (fMRI) datasets. To compare performances, we use two main criteria: First, we evaluate how well the explanations of an attribution method perform at identifying those parts of the brain whose activity we would be expect to be associated with the decoded mental states based on other empirical evidence. To this end, we compare explanations to the results of a standard general linear model (GLM; Holmes and Friston, 1998) analysis of the fMRI data as well as to the results of a meta-analysis. We find that the explanations of sensitivity analyses are generally more similar to the results of the GLM/meta-analysis when compared to the explanations of reference-based attributions and backward decompositions. Second, to understand how well the explanations capture the decision process of a DL model, we evaluate their explanation faithfulness by testing whether they correctly identify those parts of the brain whose activity is necessary for the model to accurately decode the mental states. We find that the explanations of reference-based attributions and backward decompositions are generally more faithful than those of sensitivity analyses. Last, we perform two sanity checks for attribution methods (Adebayo et al., 2018) to test whether their explanations are in fact sensitive to the characteristics of the analyzed model and data. We find that sensitivity analyses perform better overall in the sanity checks than reference-based attributions and backward decompositions.

Taken together, these findings lead us to a twofold recommendation for attribution methods in mental state decoding: If researchers want to understand the decision process of a DL model in mental state decoding, we recommend reference-based attribution methods (such as DeepLift SHAP (Lundberg and Lee, 2017), DeepLift (Shrikumar et al., 2017), and Integrated Gradients (Sundararajan et al., 2017)) because their explanations are the most faithful in our analyses while also performing well in the sanity checks. By contrast, if researchers want to understand the association between mental states and brain activity, and merely use DL models as a tool to study this association, we recommend sensitivity analyses (such as Gradient Analysis (Simonyan et al., 2014), Guided Backpropagation (Springenberg et al., 2015), and Guided GradCam (Selvaraju et al., 2017)) because their explanations align best with other empirical evidence on the association of brain activity and decoded mental states while also performing well in the sanity checks.

## 1. Methods

### 1.1. Data

We analyzed three fMRI datasets in this study, namely, the data of: i) 44 randomly-selected individuals in the motor task of the Human Connectome Project (HCP; Van Essen et al., 2013), ii) 44 randomly-selected individuals in the HCP’s working memory (WM) task, and iii) 58 individuals in a pain and social rejection experiment published by Woo et al. (2014). We refer to these three datasets respectively as “MOTOR”, “WM”, and “heat-rejection” in the following and provide a brief overview of their experiment tasks as well as details on the fMRI acquisition and preprocessing. For any further methodological details, we refer the reader to the original publications (Van Essen et al., 2013; Woo et al., 2014).

#### 1.1.1. Experiment tasks

##### Heat-rejection

The heat-rejection dataset comprises fMRI data from two experimental tasks. In the rejection task, individuals either see head shots of an ex-partner with a cue-phrase beneath the photo directing them to think about how they felt during the break-up (rejection) or a head shot of a close friend with a cue-phrase directing them to think about a specific positive experience with this friend (no rejection). In the somatic pain task, individuals focus on a hot (painful) or warm (not painful) stimulus that is delivered to their left forearm (with temperatures calibrated to each participant). Each rejection trial begins with a 7 s fixation cross, followed by a 15 s presentation period of a photo (ex-partner or friend), a 5 s five-point affect rating period, and 18 s of a visuo-spatial control task in which individuals see an arrow pointing left or right and are asked to indicate in which direction the arrow is pointing. Heat trials are identical in structure to rejection trials with the exception that individuals see a fixation cross during the 15 s thermal stimulation period (consisting of a 1.5 s temperature ramp up/down and 12 s at peak temperature) and subsequently use the five-point rating scale to report their experienced pain level.

##### MOTOR

In the HCP’s motor task, individuals see visual cues asking them to tap their left or right fingers, squeeze their left or right toes or move their tongue. The task is presented in blocks of 12 s, each including one movement type, preceded by 3 s cue. Two fMRI runs are collected for this task, each comprising two blocks of tongue movements, four blocks of hand movements (2 left, 2 right), and four blocks of foot movements (again, 2 left and 2 right) as well as three 15 s fixation blocks.

##### WM

In the HCP’s working memory task, individuals see images of one of four different stimulus types (body parts, faces, places or tools). In one half of the task blocks, individuals are asked to indicate whether the current stimulus is the same as the stimulus that was shown 2 before (2-back). In the other half of the task blocks, individuals are asked to indicate whether the currently presented stimulus is the same as a target stimulus that was shown at the beginning of the block (0-back). Two fMRI runs are collected for this task, each comprising eight task (25 s each) and four fixation (15 s each) blocks. Each task block consists of 10 trials (2.5 s each) of 2 s stimulus presentation and 0.5 s interstimulus interval. Note that we pool the data of the two N-back conditions because we are not interested in identifying any effect of the N-back condition on brain activity.

#### 1.1.2. FMRI data acquisition

##### Heat-rejection

Whole-brain EPI acquisitions were acquired on a GE 1.5 T scanner using a T2*-weighted spiral in-out sequence developed by Dr Gary Glover with TR=2,000ms, TE=40ms,flipangle=84,FOV=22cm, and 24 axial slices with 3.5×3.5×4.5mm voxels parallel to the anterior commissure-posterior commissure line (for further methodological details on fMRI data acquisition, see Woo et al., 2014).

##### Human Connectome Project

Whole-brain EPI acquisitions were acquired with a 32-channel head coil on a modified 3T Siemens Skyra with TR=720ms, TE=33.1ms,flipangle=52, in-plane FOV=20,8×18cm, and 72 slices with 2.0 mm isotropic voxels. Two fMRI runs were acquired for each task, one with a right-to-left and the other with a left-to-right phase encoding (for further methodological details on fMRI data acquisition, see Uurbil et al., 2013).

#### 1.1.3. Ethics statement

##### Heat-rejection

The study was approved by the Columbia Universitys Institutional Review Board and the University of Colorado Boulders Institutional Review Board. All participants provided written informed consent.

##### Human Connectome Project

Scanning protocols involving human participants were reviewed and approved by Washington University in St. Louiss Human Research Protection Office (HRPO), IRB 201204036. The participants provided their written informed consent to participate in the study.

#### 1.1.4. FMRI data preprocessing

##### Heat-rejection

FMRI data preprocessing was performed by the original authors (see Woo et al., 2014) and included removal of the first four volumes of each fMRI run to allow for image intensity stabilization, slice timing correction (realignment) with SPM8, spatial warping to SPMs normative atlas using warping parameters estimated from co-registered, high-resolution structural images, interpolated to 2 × 2 × 2 mm voxels, and spatial smoothing with an 8 mm FWHM Gaussian kernel.

##### Human Connectome Project

We preprocessed the fMRI data of the MOTOR and WM datasets with fmriprep 20.2.3 (Esteban et al., 2019). A detailed description of all preprocessing steps can be found in Appendix A.1.

### 1.2. BOLD maps

#### 1.2.1. Trial-level

We train DL models on trial-level voxel-wise statistical parametric maps (Friston et al., 1994) that were computed for each experimental trial of a dataset. We refer to these maps as trial-level blood-oxygen-level-dependent (BOLD) maps throughout the rest of the manuscript.

##### Heat-rejection

Trial-level BOLD maps were computed by the original authors (see Woo et al., 2014) in a GLM analysis that included boxcar regressors for each individual trial convolved with SPM8s canonical haemodynamic response function (HRF). In addition, nuisance covariates of no interest were included in the analysis representing a linear drift across time within each run, the six estimated head movement parameters for each run (x, y, z, roll, pitch and yaw; mean-centered) as well as their squares, derivatives, and squared derivatives, and indicator vectors for outlier time points as well as the first two time points of each run (for details on outlier detection, see Woo et al., 2014).

##### Human Connectome Project

We computed trial-level BOLD maps by the use of Nilearn 0.9.0 (Abraham et al., 2014) in a GLM analysis with boxcar regressors for each individual trial convolved with a standard Glover HRF (as implemented in Nilearn; leaving the fixation periods as unmodelled baselines). In addition, the analysis included nuisance regressors of no interest representing the six estimated head movement parameters (x, y, z, roll, pitch and yaw) as well as their squares, derivatives, and squared derivatives, the average signal of white matter and cerebrospinal fluid masks, the global signal, and a set of low-frequency regressors to account for slow signal drifts below 128 s.

#### 1.2.2. Subject- and group-level

To aggregate the trial-level BOLD maps to the subject- and group-level, we used a standard two-stage analysis procedure as proposed by Holmes and Friston (1998).

The subject-level analysis included a binary indicator variable for each mental state of a dataset, which we used to contrast each mental state of a dataset against all other mental states of the dataset. Note that the subject-level analysis of the two HCP datasets (WM and MOTOR) also included a binary nuisance variable for each of the two fMRI runs.

Similarly, the group-level analysis included a binary indicator variable for each subject-level contrast type (i.e., mental state) as well as a binary nuisance variable for each individual. Accordingly, the resulting group-level contrast maps correspond to a paired, two-sample t-test over the subject-level contrast maps. Note that we smoothed all subject-level contrast maps with a 5 mm FWHM Gaussian kernel in the group-level analysis.

### 1.3. Training and test data

We split each fMRI dataset into distinct training and test data by randomly designating the trial-level BOLD maps of 20% of the individuals as test data and using the maps of all remaining individuals for training. Note that we further randomly divide the training data into distinct training and validation datasets during hyper-parameter evaluation and model training (see Sections 1.5.2 and 1.5.3).

### 1.4. Meta-Analysis

To identify brain regions whose activity we would expect to be associated with each mental state included in the three datasets, beyond the results of our BOLD GLM analysis (see Section 1.2.2), we performed a meta-analysis with NeuroQuery (Docks et al., 2020). Given a text description of a mental state, NeuroQuery predicts the spatial distribution of expected whole-brain neurological observations for the described mental state. Importantly, NeuroQuery bases its predictions on the reported neural correlates of 7,547 neuroscience terms across 13,459 neuroimaging publications, which use diverse methodologies, and thereby goes beyond the results of a univariate GLM analysis of the BOLD data.

**Fig. B.1.**
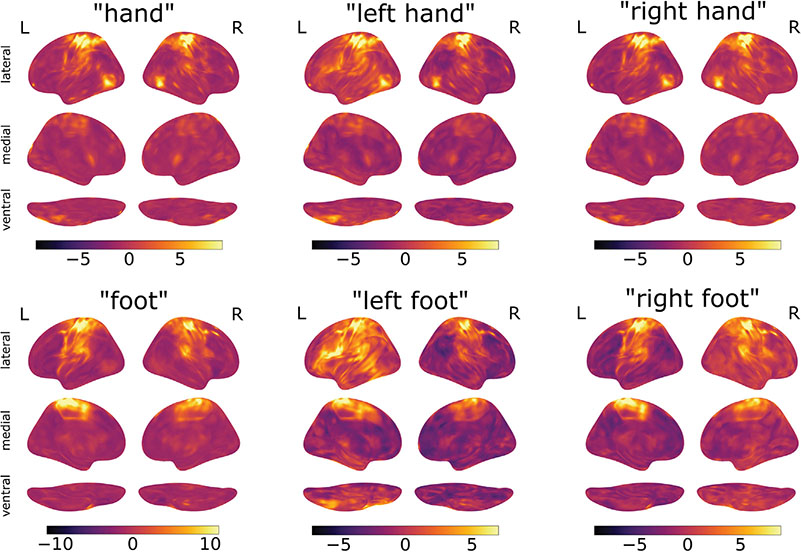
Results of a meta-analysis with NeuroQuery for the text descriptions “hand”, “left hand”, “right hand”, “foot”, “left foot”, “right foot”. As can be seen, NeuroQuery does not accurately account for the lateralization of left/right hand and foot movements. All brain maps are projected onto the inflated cortical surface of the FsAverage template (Fischl, 2012).

For this meta-analysis, we used the text descriptions “pain perception” and “social rejection” to describe the mental states of the heat-rejection dataset. For the WM dataset, we used the text descriptions “body images”, “face perception”, “place perception”, and “tool images”. For the MOTOR dataset, we aggregated over the left and right conditions of “hand” and “foot” movements by using the text descriptions “foot”, “hand”, and “tongue movement” because we found that NeuroQuery does not accurately capture the lateralization of motor brain function when using the text descriptions “left hand”, “right hand”, “left foot”, and “right foot” (see Appendix Fig. B.1). Consequently, we compare any analysis results of left/right hand/foot movements to the respective aggregate “hand” and “foot” meta-analysis results.

#### 1.5. Deep learning model

We use 3D-convolutional neural network architectures (3D-CNNs; LeCun and Bengio, 1998) as mental state decoding models, which are composed of a stack of 3D-convolution layers and a dense output layer.

A 3D-convolution layer consists of a set of 3D-kernels that each learn specific features of an input volume x. Each kernel k learns a volumetric feature that is convolved over the input, resulting in an activation map $A^{k}$ that indicates the presence of the learned feature for each spatial location of the input: $A_{i,j,l}^{k}=g\left(\sum_{m}\;\sum_{n}\;\sum_{z}\;w_{m,n,z}^{k}x_{i+m-1,j+n-1,l+z-1}+b^{k}\right)$. Here, $b^{k}$ and $w^{k}$ represent the bias and weights of the kernel, while g represents the rectified linear unit activation function: g(x)=max(0,x). The indices m, n, and z indicate the row, column, and height of the 3D-convolution kernel, while i, j, and l indicate the coronal (i.e., row), saggital (i.e., column), and axial (i.e., height) dimension of the activation map $A^{k}$. The models move all convolution kernels over their input at a stride size of 2, thus applying the kernels to every other value of a layer’s input. The models further apply batch-normalization (Ioffe and Szegedy, 2015) to the linear outputs of each convolution layer (before the non-linear activation).

To make a decoding decision, the models flatten the activation maps A resulting from the last convolution layer and pass the flattened maps to a dense softmax output layer to predict a probability $p_{c}$ that input x represents mental state $c:p_{c}=\sigma \left(b_{c}+\sum_{i}a_{i}w_{ic}\right)$, where b and w represent the layer’s bias and weights, while σ indicates the softmax function: $\sigma (x{)}_{j}=\frac{e^{x_{j}}}{\sum_{i}e^{x_{i}}}$.

#### 1.5.1. Optimization

We optimize DL models to correctly decode the mental state associated with each trial-level BOLD map (namely, “heat” and “rejection” for the heat-rejection dataset, “left foot (lf)”, “right foot (rf)”, “left hand (lh)”, “right hand (rh)”, and “tongue (t)” for the MOTOR dataset, and “body”, “faces”, “places”, and “tools” for the WM dataset; see Section 1.1.1), using a standard cross-entropy loss objective: $L=-\sum_{c}\;y_{c}log\;p_{c}\;(y_{c}$ indicates a binary variable that equals 1 if c is the correct mental state and 0 otherwise).

By the use of this objective, we optimize models with the ADAM algorithm (Kingma and Ba, 2017) for a minimum of 10 and a maximum of 100 training epochs, where each epochs represents an entire iteration over the training data. We stop training if one of the following two early stopping criteria is met after an initial grace period of 10 epochs: i) the recorded evaluation loss is worse than its previous best for three evaluations (we always evaluate at the end of an epoch) or ii) the standard deviation of the last 10 recorded evaluation losses does not exceed 0.01.

#### 1.5.2. Hyper-parameter selection

For each dataset, we perform a grid search to determine a set of best-performing model and optimization hyper-parameters. Specifically, we search over the number of convolutional layers (3 and 4), the number of kernels per layer (4, 8, 16, ad 32), and the kernels’ size (3 and 5) used for the model as well as the mini-batch size (32 and 64), learning rate $\left(3e^{-4}\right.$ and $\left.1e^{-3}\right)$, and dropout rate (0%, 25%, and 50%; applied to convolution layers) used for optimization. We evaluate the performance of each of the resulting 192 parameter configurations in a three-fold cross-validation procedure, using each dataset’s training data (see Section 1.3). We determine the best-performing configuration for each dataset by computing each configuration’s mean decoding error ϵ (defined as 1 minus the fraction of correctly decoded trial-level BOLD maps) in the training and validation data over the three folds $\left(\epsilon^{T}\right.$ and $\epsilon^{V}$ respectively) and assigning a performance score $\lambda_{i}$ to each configuration $i:\lambda_{i}=\epsilon_{i}^{V}+\left|\epsilon_{i}^{V}-\epsilon_{i}^{T}\right|$. This score quantifies how well a model performed in the validation data when also accounting for the difference in model performance to the training data. Accordingly, we define the best performing configuration for each dataset as the one with the lowest $\lambda_{i}$ and use this configuration for all further analyses.

#### 1.5.3. Final model fits

A wealth of recent empirical work has shown that DL model performances are strongly dependent on many non-deterministic aspects of their training, such as random weight initializations and random shufflings of the data during training (Henderson et al., 2018; Lucic et al., 2018; Thomas et al., 2022a). For this reason, we perform 10 training runs with the best-performing model configuration of each dataset (see Section 1.5.2), using different random seeds and random splits of the data into distinct training and validation datasets (comprising 90% and 10% of the trial-level BOLD maps respectively) for each run. If not reported otherwise, we use the model that achieved the best evaluation loss during training for all further analyses.

### 1.6. Attribution approaches

We assume that the analyzed model represents some function f mapping an input $x\in R^{N}$ to some output f(x)∈R, such that $f:R^{N}\to R$. In the following, we present a set of attribution methods $\eta_{f}$ that seek to provide insights into this mapping between x and f(x), which is specific to each input and trained model. Specifically, these methods attribute a relevance score $r_{n}\in R$ to each input feature n=1,…,N, quantifying the feature’s contribution to f(x), such that: $\eta_{f}:R^{n}\to R^{n}$

**Fig. 1.**
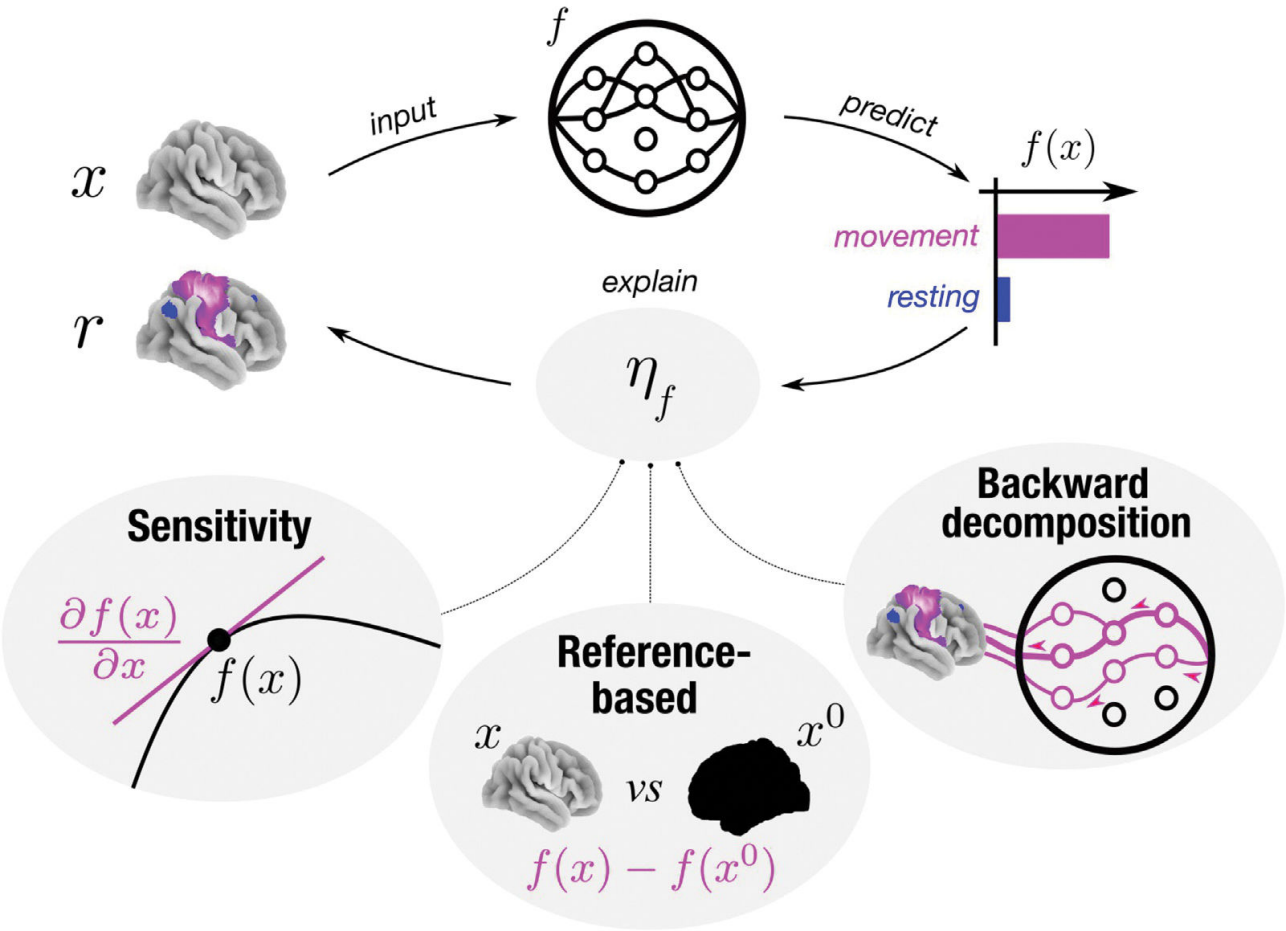
Explanation approaches.All considered explanation approaches $\eta_{f}$ seek to explain a mental state decoding decision f(x) of model f by attributing a relevance r to each feature of the input x for the model’s decision. Backward decompositions attribute relevance to input features by decomposing the decoding decision in a backward pass through the model into the contributions of lower-level model units to the decision, up to the input space, where a contribution for each input feature can be defined. Reference-based attributions attribute relevance to input features by comparing the model’s response to a given input to its response to a reference input $x^{0}$ (often chosen to be neutral). Sensitivity analyses attribute relevance to input features according to how sensitively a model’s decoding decision responds to an input feature’s value (e.g., as measured by the partial derivative $\frac{\partial f(x)}{\partial x}$).

On a high level, the presented attribution methods can be divided into sensitivity analyses, reference-based attributions, and backward decompositions (see Fig. 1). Sensitivity analyses quantify r by determining how sensitively f(x) responds to x. Reference-based attributions, by contrast, determine r by contrasting the models response to a given input x to its response to a reference input b. Backward decompositions, on the other hand, quantify r by sequentially decomposing the model’s output f(x) in a backward pass through the model into the contributions of the lower-layer model units to the predictions, until the input space is reached and a contribution for each input feature can be defined.

#### Gradient Analysis

(Simonyan et al., 2014; Zurada et al., 1994) represents the most commonly used type of sensitivity analysis and defines $r_{n}$ as the partial derivative of f(x) with respect to the input $x_{n}$, such that: $r_{n}=|\frac{\partial f(x)}{\partial x_{n}}|$. Relevance is thus assigned to those feature values to which f(x) responds most sensitively.

#### SmoothGrad

(Smilkov et al., 2017) represents an extension of Gradient Analysis to account for sharp fluctuations of the gradient $\nabla f(x)=\frac{\partial f(x)}{\partial x}$ at small scales, which can otherwise lead to noise in the resulting attributions. Specifically, SmoothGrad randomly draws K samples (we set K=50) from the neighborhood of x, by adding random Gaussian noise $𝒩\left(0,\sigma^{2}\right)$ with standard deviation σ (we set σ=1) to x, and averages the resulting absolute partial derivatives for each random sample to obtain relevances $r:r=\frac{1}{K}\sum_{1}^{K}\;\left|\nabla f\left(x+𝒩\left(0,\sigma^{2}\right)\right)\right|$.

#### InputXGradient

(Shrikumar et al., 2017) represents another extension of Gradient Analysis, which multiplies the gradient ∇f(x) by x, such that: r=∇f(x)×x, where × indicates the element-wise product. The intuition behind this approach comes from linear models, where the product of input and model coefficient (here represented by the gradient) corresponds to the total contribution of the associated feature to the model’s output.

#### Guided Backpropagation

(Springenberg et al., 2015) represents an adaptation of Gradient Analysis tailored to CNN models that primarily use ReLU Agarap (2019) activation functions. It overrides gradients of ReLU activation functions in the computation of the gradient ∇f(x) such that only non-negative gradients are backpropagated.

#### Guided Gradient-weighted Class Activation Mapping (Guided GradCam)

(Selvaraju et al., 2017) represents another type of sensitivity analysis tailored to CNNs that combines Guided Backpropagation with the GradCam (Selvaraju et al., 2017) technique. Specifically, GradCam first computes the gradient ∇f(x) with respect to the feature maps $A^{k}\in R^{D}$ of the last convolutional layer L (closest to the model’s output) and then global-average pools the resulting gradients to obtain an importance weight $\alpha_{k}$ for each kernel $k\in L:\alpha_{k}=\frac{1}{D}\sum_{d=1}^{D}\;\frac{\partial f(x)}{\partial A_{d}^{k}}$. Conceptually, $a_{k}$ captures the importance of feature map $A^{k}$ for the decoded mental state. Next, GradCam uses the importance weights $\alpha_{k}$ to combine the activation maps $A^{k}$ to an aggregate heatmap of relevances $r_{A}:r_{A}=\sigma (\sum_{k}\;\alpha_{k}A^{k})$, where σ represents the ReLU function (σ(x)=max(0,x)). Last, to obtain relevances r, Guided GradCam upsamples the relevances $r_{A}$ to the original input dimension and multiplies the upsampled maps with the relevance attributions of an application of Guided Backpropagation to f(x) (see above).

#### Integrated Gradients

(Sundararajan et al., 2017) represents a reference-based attribution method that assigns relevance r by integrating the gradient ∇f(x) along a linear trajectory in the input space, connecting the current input x to some neutral reference input $b:r^{b}=(x-b)\int_{\alpha =0}^{1}\;\frac{\delta f(b+\alpha (x-b))}{\delta x}$. Integrated Gradients thus assigns relevance to input features according to how much the models output changes when these features are scaled from the reference value to their current value. In our analyses, we chose two reference inputs, namely, an all-zero input $b^{0}$ (as recommended in Sundararajan et al., 2017) as well as an average over all inputs in the analyzed dataset $b^{\mu }$, and averaged over the two resulting attributions to obtain relevance values $r:r=0.5r^{0}+0.5r^{\mu }$.

#### Deep Learning Important FeaTures (DeepLift) DeepLift

(Shrikumar et al., 2017) represents another type of reference-based attribution method. Similar to Integrated Gradients, DeepLift determines relevances r by comparing model responses for a given input x to the model’s response to some neutral reference input b. To this end, DeepLift defines a contribution score $C_{\Delta x_{n}\Delta f(x)}$, describing the difference-from-reference response Δf(x)=f(x)−f(b) that is attributed to a difference-from-reference input Δx=x−b. Note that $\sum_{n=1}^{N}\;C_{\Delta x_{n}\Delta f(x)}=\Delta f(x)$. To compute these contribution scores, DeepLift uses multipliers $m_{\Delta x_{n}\Delta f(x)}$ that are defined as $m_{\Delta x\Delta f(x)}=\frac{C_{\Delta x\Delta f(x)}}{\Delta x}$ and thereby quantify the contribution of Δx to Δf(x) scaled by Δx. For any unit $x^{\left(l\right)}$ in model layer l and any unit $x^{\left(l-1\right)}$ in the preceding layer l−1, these multipliers can be computed as: $m_{\Delta x_{n}^{\left(l-1\right)}\Delta f(x)}=\sum_{j}m_{\Delta x_{n}^{\left(l-1\right)}\Delta x_{j}^{\left(l\right)}}m_{\Delta x_{j}^{\left(l\right)}\Delta f(x)}$, where $\Delta x_{j}^{\left(l\right)}$ indicates the difference in input feature j of layer l to its value for the reference input, in line with the chain rule. Relevance $r_{n}$ for input feature n can then be obtained as: $r_{n}=C_{\Delta x_{n}\Delta f(x)}=\Delta x_{n}m_{\Delta x_{n}\Delta f(x)}$. In its basic formulation, DeepLift uses two rules to compute contribution scores: The linear rule applies to dense and convolution layers, which compute $a=w^{0}+\sum_{n}w_{n}x_{n}$, where $w^{0}$ and w indicate bias and weights and x the input, and defines $C_{\Delta x_{n}\Delta a}=\Delta x_{n}w_{n}$ and accordingly $m_{\Delta x_{n}\Delta a}=\frac{C_{\Delta x_{n}\Delta a}}{\Delta x_{n}}$. The rescale rule applies to all non-linear transformations σ(a) (e.g., ReLU or sigmoid functions) and defines $C_{\Delta x\Delta \sigma (a)}=\Delta \sigma (a)$ and accordingly $m_{\Delta x\Delta \sigma (a)}=\frac{C_{\Delta x\Delta \sigma (a)}}{\Delta x}$.

#### DeepLift SHapley Additive exPlanation (DeepLift SHAP)

(Lundberg and Lee, 2017) represents another type of reference-based attribution method that combines DeepLift with SHAP, a method for computing Shapley values (Shapley, 1952) for a conditional expectation function of the analyzed model. Specifically, SHAP values attribute to each input feature the change in expected model prediction conditioned on a feature of interest. To approximate SHAP values using DeepLift for a given input x, DeepLift SHAP draws K (we set K=50) random samples from the data (to approximate the set of other possible feature coalitions) and averages over the DeepLift attributions for each random sample when treating the random sample as a reference input b.

#### Layer-wise relevance propagation (LRP)

(Bach et al., 2015) represents a backward decomposition method. Let i and j be the indices of two models units in successive model layers l and l+1 and $r_{j}^{\left(l+1\right)}$ the relevance of unit j for f(x). To redistribute relevance between layers, several rules have been proposed (Bach et al., 2015; Kohlbrenner et al., 2020; Montavon et al., 2019), which generally follow from ${r_{i}^{\left(l\right)}=\sum }_{j}\frac{a_{i}w_{ij}}{\sum_{i}a_{i}w_{ij}}r_{j}^{\left(l+1\right)}$, where a and w represent the input and weight of model unit i in layer l. Importantly, LRP conserves relevance between layers, such that: $\sum_{n}\;r_{n}=\sum_{i}\;r_{i}^{\left(l\right)}=\sum_{j}\;r_{j}^{\left(l+1\right)}=f(x)$. In line with the recommendations by Montavon et al. (2019), we use a composite of relevance redistribution rules, namely, the LRP-0 rule $\left(r_{i}^{\left(l\right)}=\sum_{j}\frac{a_{i}w_{ij}}{\sum_{i}a_{i}w_{ij}}r_{j}^{\left(l+1\right)}\right)$ for the dense output layer and the LRP-γ rule ($r_{i}^{\left(l\right)}=\sum_{j}\frac{a_{i}(w_{ij}+\gamma w_{ij}^{+})}{\sum_{i}a_{i}(w_{ij}+\gamma w_{ij}^{+})}r_{j}^{\left(l+1\right)}$, where γ controls the positive contributions to r (we set γ=0.25)) for all convolution layers.

#### 1.6.1. Implementation

We implement all attribution methods using the Captum Python library (Kokhlikyan et al., 2020), developed and maintained by Meta Open Source, with the exception of LRP, which relies on a custom implementation provided by the Zennit Python library (Anders et al., 2021) that is developed and maintained by the original LRP authors.

#### 1.6.2. Attribution maps

With each of the considered attribution methods, we attribute the mental state decoding decisions of each of the 10 trained instances of each selected model configuration per dataset (see Section 1.5.2) for its respective test trial-level BOLD maps (see Section 1.3). Importantly, we always interpret the models’ decoding decision for the actual mental state associated with each BOLD map. This analysis results in a dataset of 10 attribution maps (one per model instance) per trial-level BOLD map per attribution method.

To aggregate these data over the 10 model instances, and thereby account for any non-deterministic training effects on the attributions, we perform a standard two-stage GLM analysis by first computing a subject-level GLM analysis of the trial-level attribution maps and subsequently aggregating the subject-level attribution maps in a random-effects group-level analysis. We use the same GLM procedure as for the BOLD data (see Section 1.2.2), with the exception that the subject-level analysis includes additional binary nuisance variables for the 10 model training runs and a nuisance variable indicating sum of the attribution values of each trial-level attribution map (as attribution sums can vary between decoding decisions, e.g., due to varying model decoding certainty).

### 1.7. Evaluating attributions

We evaluate the quality of the attribution methods’ explanations in three analyses (see Sections 1.7.1, 1.7.2, and 1.7.3): First, we test how well the explanations align with other empirical evidence on the association between the decoded mental states and brain activity by comparing the attribution maps (see Section 1.6.2) to the results of a standard GLM analysis of the BOLD data (see Section 1.2.2) as well as to the results of a meta-analysis (see Section 1.4). Second, we evaluate how accurately the explanations capture the decision process of a model by estimating their faithfulness. Third, we perform two sanity checks for attribution methods to test whether their explanations are in fact sensitive to the analyzed model and data.

#### 1.7.1. Alignment with other empirical evidence

To test whether the explanations of an attribution method identify those parts of the brain whose activity we would expect to be associated with the decoded mental states based on other empirical evidence, we compare the explanations to the results of a GLM analysis of the BOLD data (see Section 1.2.2) as well as to the results of a meta-analysis for each mental state (see Section 1.4).

To quantify the similarity of the attribution and GLM/meta-analysis maps, we compute their mutual information (Kraskov et al., 2004). On a high level, mutual information measures the amount of information about one variable (here, one brain map) that can be gained by observing another variable (here, the other brain map). Unlike linear measures of association, such as correlation, mutual information contains information about all dependence between two variables, linear and nonlinear. Formally, mutual information I between two discrete random variables X and Y, whose joint probability distribution is $P_{XY}(x,y)$, is given by: $I(X;Y)=\sum_{x\in X}\;\sum_{y\in Y}\;P_{XY}(x,y)log\frac{P_{XY}(x,y)}{P_{X}(x)P_{Y}(y)}$, where $P_{X}(x)$ and $P_{Y}(y)$ are the marginals: $P_{X}(x)=\sum_{y\in Y}\;P_{XY}(x,y)$. In our case, $X\in R^{N}$ and $Y\in R^{N}$ each represents a vector of all N voxel values contained in one of the two brain maps. As these voxel values are on a continuous scale, and not discrete, we use an estimate of mutual information for continuous variables implemented by the scikit-learn Python library (Pedregosa et al., 2011), which estimates mutual information through non-parametric methods based on entropy estimation from k-nearest neighbor distances (Kraskov et al., 2004; Ross, 2014). Specifically, mutual information I is here estimated as: $I(X;Y)=\psi (N)+\psi (k)-\frac{1}{N}\sum_{x\in X}\;\psi \left(N_{x}\right)-\frac{1}{N}\sum_{y\in Y}\;\psi \left(N_{y}\right)$, where ψ indicates the digamma function, N the number of brain map voxel values, k the number of nearest neighbors (we set k=3 as recommended by scikit-learn), and $N_{x}$ and $N_{y}$ indicate the number of data points within the k-nearest neighbor radius of voxel values x∈X and y∈Y respectively.

We chose mutual information as a similarity measure because the associations between the maps resulting from the GLM/meta-analysis and attributions do not need to be linear. For example, it is possible that a DL model learns to identify a mental state through the activity of voxels that are meaningfully more active in this state as well as the activity of voxels that are meaningfully less active in this state, resulting in an attribution map that assigns high relevance to voxels that exhibit both positive and negative values in a GLM analysis (as shown by Thomas et al., 2021). An example of such a case is provided in Fig. 3 where the attribution maps of DeepLift SHAP, Gradient Analysis, and SmoothGrad assign high positive relevance to some of the voxels in the occipital cortex that received high negative values by a GLM analysis of the BOLD data.

We also chose mutual information as a similarity measure because it is a symmetric measure of similarity and thereby accounts for cases in which either the attribution or reference map (i.e., of the GLM/meta-analysis) highlight voxels that the respective other does not. In a conventional univariate analysis of fMRI data, such as with the GLM, the activity pattern of each voxel is individually tested for its association with a target variable (e.g., a mental state). DL models trained on whole-brain fMRI data, by contrast, look for patterns in the activity of all brain voxels that are associated with the target variable. Accordingly, it is possible that DL models learn that the activity patterns of certain voxels are redundant in their predictive information about the mental states, because of the generally high spatial correlation of brain activity, resulting in attribution maps that only include a subset of those voxels identified by a univariate analysis of the data. An example of such a case is provided in Fig. 3 where a GLM analysis of the BOLD data assigns high positive values to voxels in the superior temporal gyrus and parts of the supramarginal gyrus and precuneus, whereas the attribution maps of DeepLift, DeepLift SHAP, Integrated Gradients, LPR, and InputXGradient do not highlight these parts of the brain. Similarly, it is possible that DL models learn that the activity of some voxels is solely predictive of a mental state in conjunction with the activity of some other voxels (a key assumption of multi-voxel pattern analysis Norman et al., 2006), resulting in an attribution map that assigns high relevance to voxels that are not identified by a univariate analysis of the data.

#### 1.7.2. Explanation faithfulness

An explanation can generally be considered as being faithful if it correctly identifies those features of the input that are most relevant for the model’s decoding decision (Samek et al., 2021). Accordingly, we test whether removing the features (i.e., voxel values) from the input that received the highest relevance by an attribution method affects the model’s ability to correctly decode the mental states. We remove voxel values by setting them to 0. To quantify faithfulness, we repeat this analysis for different occlusion rates, from 0% (indicating no occlusion) to 50% (indicating that those voxels are occluded that received the highest 50% of relevance values), and record the occlusion rate at which the model’s decoding accuracy first drops to chance level for every repetition (indicating that all information has been removed from the data that the model relies on to accurately identify the mental states). If an attribution method has high explanation faithfulness, model decoding accuracies should drop to chance level at lower occlusion rates when compared to methods with lower explanation faithfulness.

#### 1.7.3. Sanity checks

We perform two sanity checks for attribution methods, as recently proposed by Adebayo et al. (2018), to test the overall scope and quality of their explanations. Specifically, Adebayo et al. (2018) propose two tests to probe whether the explanations of an attribution method are specific to the tested model and data by testing how much the explanations change when the method is applied to a model with the same architecture but random weights (the model randomization test) and a model trained on a version of the data with randomly permuted labels (the data randomization test). If the explanations are dependent on the specific parameters of the model, they should differ between the originally trained model and the model variant with random weights. If the explanations are similar, however, the attribution method can be considered insensitive to the studied model and therefore not well-suited to capture the models’ decision process. Similarly, if the explanations of an attribution method account for the labeling of the data, it should produce different explanations for the model trained on the original dataset and a model variant trained on a version of the data with randomly shuffled labels. If the explanations are similar, however, the attribution method can be considered insensitive to the labeling of the data and therefore not well-suited to understand the model’s learned mapping between these labels and the data. We implement the model randomization test by first creating an instance of each dataset’s model configuration with randomized weights. We then attribute the test mental state decoding decisions of this randomized model variant with each attribution method and compare the resulting attributions to those of the original (not randomized) models. Similarly, we implement the data randomization test by first training an instance of each model configuration on a version of the training data with randomized mental state labels and then comparing the attributions for this model, which has learned to memorize the mapping between random labels and data, to those of the original model that was trained on the original (not randomized) training data. As before, we use mutual information to quantify the similarity of attribution maps (see Section 1.7.1).

#### 1.7.4. Statistical testing

To statistically test for differences between attribution methods on any evaluation metric, we estimate linear regression models that include one binary indicator variable for all attribution methods other than DeepLift, which we treat as unmodelled baseline (if not reported otherwise). We fit regression models in a Bayesian framework by the use of the Bayesian Model-Building Interface (bambi 0.9.0; Capretto et al., 2022) and by sampling four chains per parameter (5,000 samples per chain after 5,000 discarded tuning samples) using the Markov chain Monte Carlo No-U-Turn-Sampler (NUTS; Hoffman and Gelman, 2014) with bambi’s automatically generated priors. We determine a method as meaningfully different from DeepLift (the baseline) on the analyzed metric if the estimated 94% highest-density interval of the method’s coefficient does not include 0. All posterior traces are checked for convergence according to the GelmanRubin statistic $\left(|\overset{ˆ}{R}-1|<.01\right)$.

### 1.8. Data and code availability statement

#### Data

FMRI data for the MOTOR and WM datasets were provided by the Human Connectome Project (HCP S1200 release^1^), WU Minn Consortium (Principal Investigators: David Van Essen and Kamil Ugurbil; 1U54MH091657) funded by the 16 NIH Institutes and Centers that support the NIH Blueprint for Neuroscience Research; and by the McDonnell Center for Systems Neuroscience at Washington University. Trial-level BOLD maps for the heat-rejection dataset were publicly shared by Kohoutov et al. (2020).^2^ No experimental activity involving the human participants took place at the authors institutions. Only de-identified, publicly released data were used in this study.

#### Code

All code required to reproduce the results of our analyses are available at: https://github.com/athms/xai-brain-decoding-benchmark.

#### Software

All analyses were performed in Python 3.9, using the PyTorch 1.10.2 (Paszke et al., 2019), Weights and Biases 0.12.11 (https://www.wandb.com), Captum 0.5.0 (Kokhlikyan et al., 2020), Nilearn 0.9.0 (Abraham et al., 2014), Seaborn 0.11.2 (Waskom, 2021), Ray Tune 0.13.0 (Liaw et al., 2018), Zennit 0.4.5 (Anders et al., 2021), Numpy 1.19.5 (Oliphant, 2015), Scipy 1.9.0 (Virtanen et al., 2020), Matplotlib 3.3.4 (Hunter, 2007), Bambi 0.9.0 (Capretto et al., 2022), and Arviz 0.12.1 (Kumar et al., 2019) libraries.

## 2. Results

### 2.1. Models accurately decode mental states

**Fig. 2.**
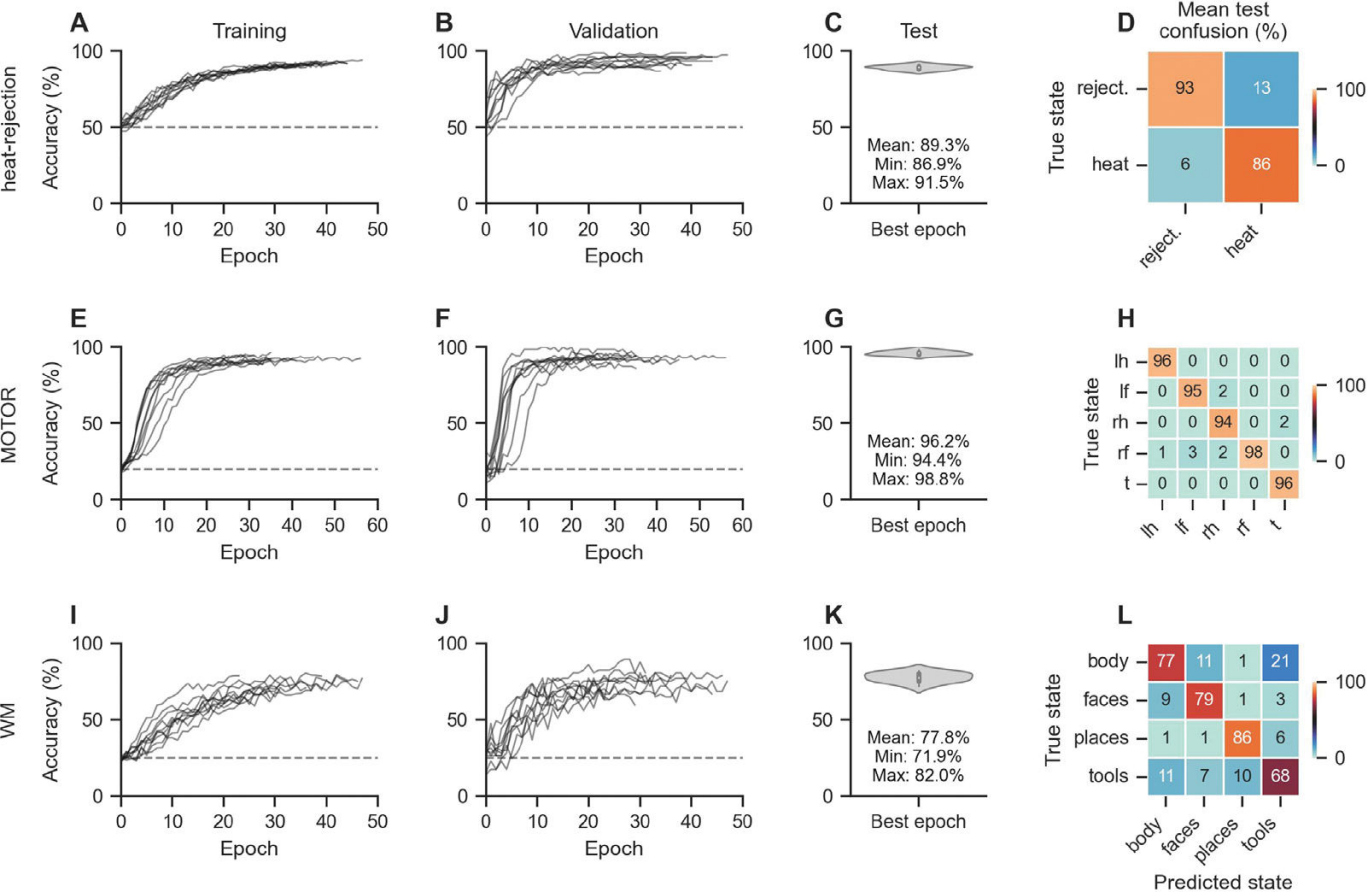
Decoding performance.For each dataset, we train 10 instances of the best-performing model configuration (see Table 1 and Section 1.5.3). We solely vary the random seed and training/validation data split between training runs. A-B,E-F,I-J: The models perform well in decoding the mental states from the trial-level BOLD maps in the training (A,E,I) and validation datasets (B,F,J). Lines indicate decoding accuracies of individual model training runs. Dashed horizontal lines indicate chance accuracies. C-D,G-H,K-L: The trained models also perform well in decoding the mental states of the left-out test datasets (C,G,K) with overall low average confusion rates (D,H,L). Violin plots show the distribution of test decoding accuracies across training runs.

After determining a set of best-performing model and optimization configurations for each dataset (see Section 1.5.2), we train 10 model instances according to these configurations on the training data of each dataset, using different random seeds and training/validation splits of the training data between runs (to account for any non-deterministic training effects; see Section 1.5.3). The models learned well over the course of their training (Fig. 2 A–B,E–F,I–J), with average test decoding accuracies of 89.3% [86.9%, 91.5%] (heat-rejection), 96.2% [94.4%, 98.8%] (MOTOR), and 77.8% [71.9%, 82.0%] (WM) (reported as mean [min, max]; Fig. 2 C,G,K). We also computed average test confusion rates over the 10 training runs per dataset (Fig. 2 D,H,L) and found that the models generally exhibited little confusion between mental states (with the exception of the body and tools states of the WM dataset; Fig. 2 L).

### 2.2. Explanations of sensitivity analyses align better with other empirical evidence

As the trained models performed well in decoding the mental states (see Fig. 2), we proceeded to attribute their test decoding decisions with each considered attribution method (for details, see Section 1.6.2).

**Fig. 3.**
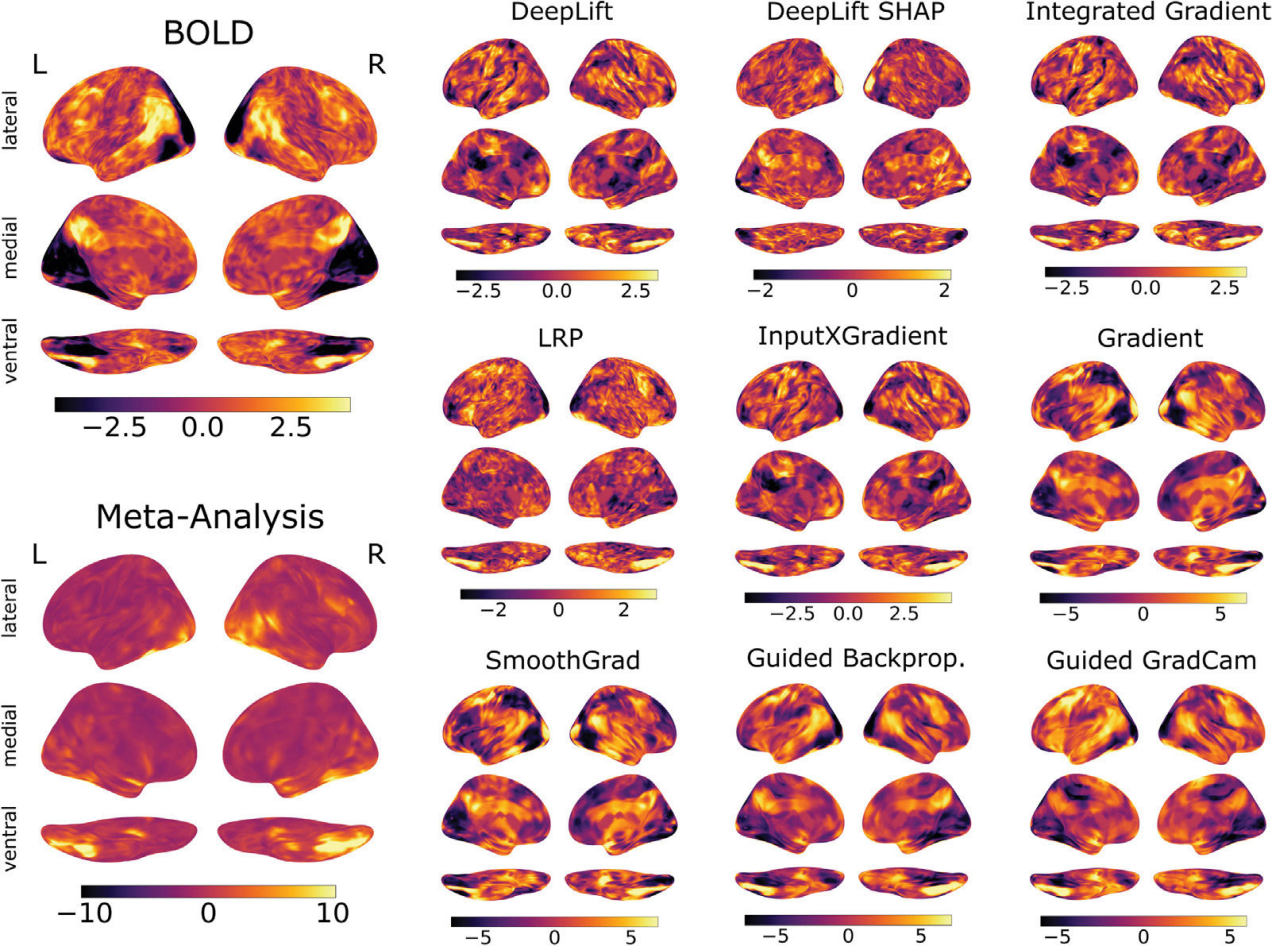
Brain maps for the “faces” state of the WM dataset. Brain maps indicate the results of a two-stage random effects analysis of the trial-level BOLD maps (upper left) as well as the test data attributions of each attribution method. Brain maps show the resulting Z-scores of a contrast between the “face” state and all other mental states of the WM dataset. We also performed a meta-analysis with NeuroQuery for the term “face perception” (lower left). All brain maps are projected onto the inflated cortical surface of the FsAverage template (Fischl, 2012).

Figure 3 provides an overview of the group-level attribution maps for the “faces” state of the WM dataset, in which individuals view images of faces, next to the results of a corresponding group-level GLM and meta-analysis (for details, see Sections 1.2.2 and 1.4). For this mental state, the majority of attribution methods correctly attributed high relevance to those voxels in the fusiform face area (FFA; Heekeren et al., 2004) that also showed a positive association with this state in the GLM/meta-analysis (with the exception of DeepLift SHAP whose attribution map only highlights a small subset of those FFA voxels). Note that the GLM analysis further indicates a positive association of this mental state with the activity of the upper parts of the superior temporal gyrus and parts of the supramarginal gyrus and precuneus. These additional activations, beyond the FFA, are also indicated in the attribution maps of Gradient Analysis, SmoothGrad, and Guided Backpropagation (and in part also for Guided GradCam), all types of sensitivity analysis. However, these additional activations are not present in the attribution maps of the the considered backward decomposition and reference-based attribution methods (with the exception of DeepLift SHAP whose attribution map also assigns some relevance to the precuneus).

As can be seen, it is generally difficult to discern how the attribution maps compare in their alignment with the results of the GLM/meta-analysis by visual inspection alone. For this reason, we next took a quantitative approach to answering this question by quantifying the similarity of the attribution maps to the results of the GLM/meta-analysis by means of mutual information (for details, see Sections 1.7.1).

**Fig. 4.**
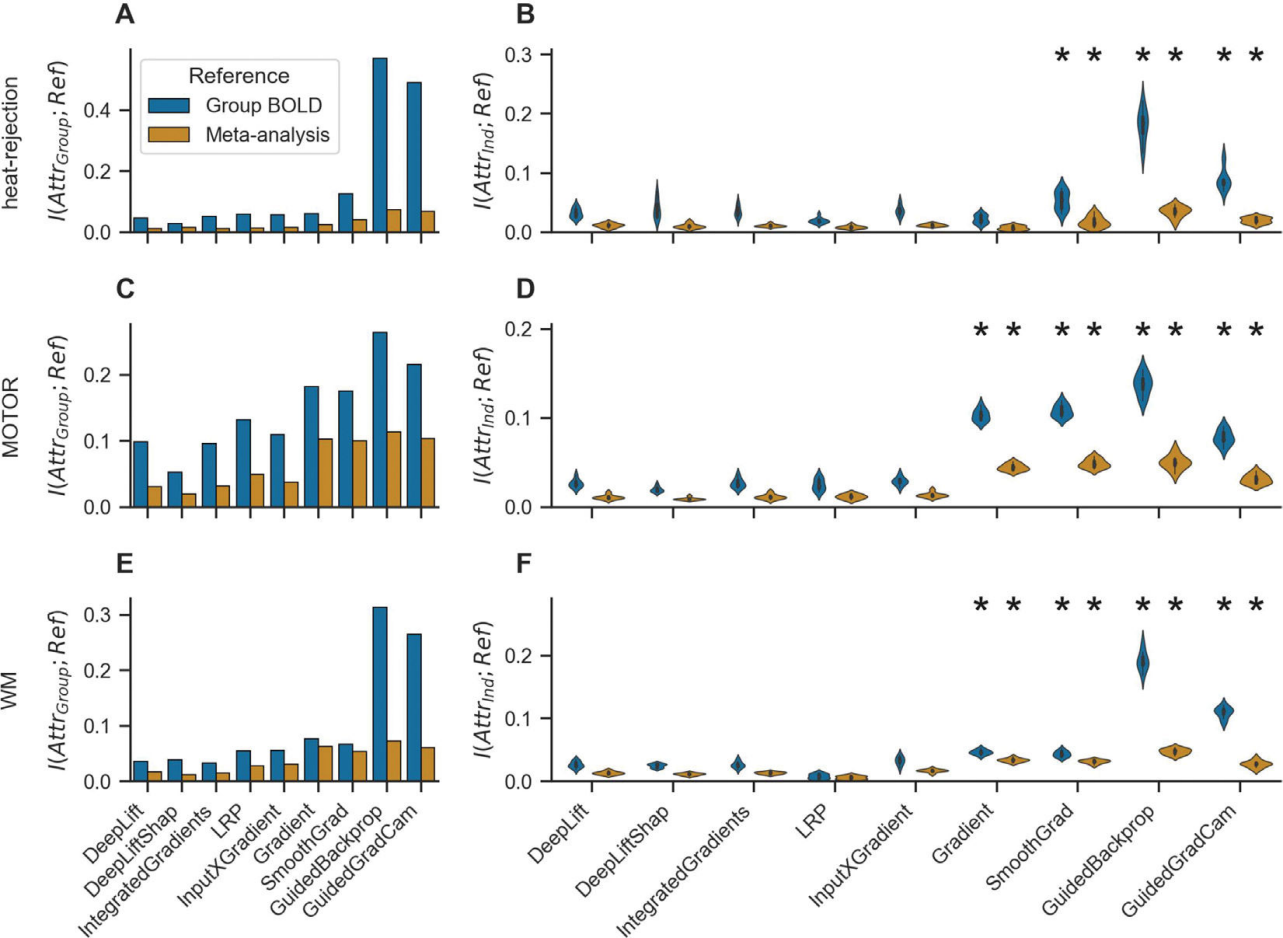
Alignment of explanations with other empirical evidence. To test whether the attribution maps accurately identify those parts of the brain whose activity we would expect to be associated with the decoded mental states based on other empirical evidence, we compute their mutual information with the results of a standard GLM analysis of the BOLD data (blue) as well as with the results of a meta-analysis with NeuroQuery (yellow). A,C,E: The group-level maps of Guided Backpropagation and Guided GradCam exhibit higher mutual information (i.e., similarity) with the GLM/meta-analysis maps than the group-level maps of the other attribution methods. Albeit lower, the group-level maps of Gradient Analysis and SmoothGrad also exhibit overall higher similarity scores than the group-level maps of the remaining attribution methods. Bar heights indicate mean mutual information over the mental states of a dataset. B,D,F: The subject-level maps of Guided Backpropagation, Guided GradCam, Gradient Analysis, and SmoothGrad are more similar to the GLM/meta-analysis maps than the subject-level maps of the other attribution methods. Violin plots show the distribution of mean mutual information per individual in the test data. Black stars indicate that the distribution of subject means of an attribution method are meaningfully larger than for DeepLift (for details, see Section 1.7.4). Colors indicate the reference map used for the comparison. (For interpretation of the references to colour in this figure legend, the reader is referred to the web version of this article.)

This analysis revealed that the group-level attribution maps of Guided Backpropagation and Guided GradCam, two types of sensitivity analysis, are more similar to the group-level BOLD and meta-analysis maps than those of the other attribution methods (as indicated by higher mutual information scores; Fig. 4 A,C,E). We also found that the group-level maps of Gradient Analysis and SmoothGrad, again two types of sensitivity analyses, exhibit less, but still comparably high, similarity to the group-level BOLD and meta-analysis maps when compared to the remaining attribution methods (Fig. 4 A,C,E).

As the trained models can draw from their knowledge about the group of subjects in their training data when decoding the trial-level BOLD maps of individual subjects, we also tested how well the subject-level attribution maps of each attribution method align with the group-level BOLD and meta-analysis maps. This analysis showed that the subject-level attribution maps of Guided Backpropagation, Guided GradCam, SmoothGrad, and Gradient Analysis, all variants of sensitivity analysis, are generally more similar to the group-level BOLD and meta-analysis maps than the subject-level attribution maps of the other attribution methods (Fig. 4 B,D,F).

**Fig. B.2.**
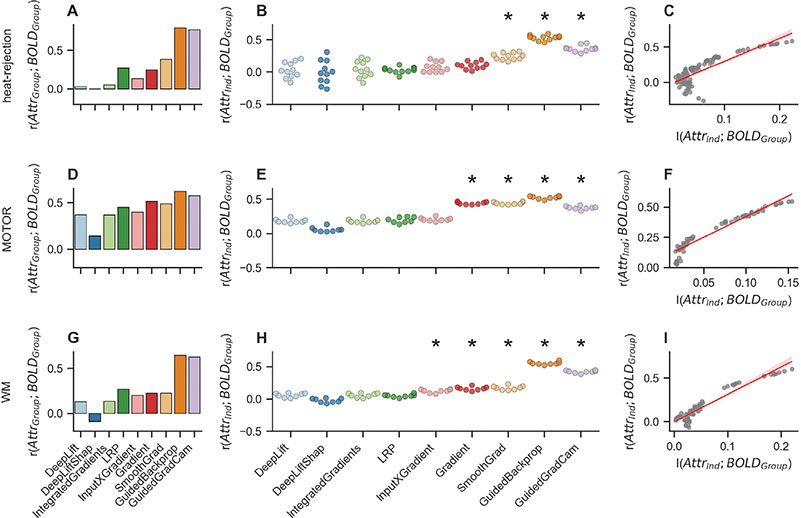
Similarity of BOLD and attribution maps as estimated by Pearson correlation. A-B,D-E,G-H: Replication of respective parts of Fig. 4 of the main text using Pearson correlation as similarity measure. C,F,I: Pearson correlation scores are strongly correlated with corresponding mutual information scores. Red lines indicate linear regression fit surrounded by 95% confidence intervals.

**Fig. B.3.**
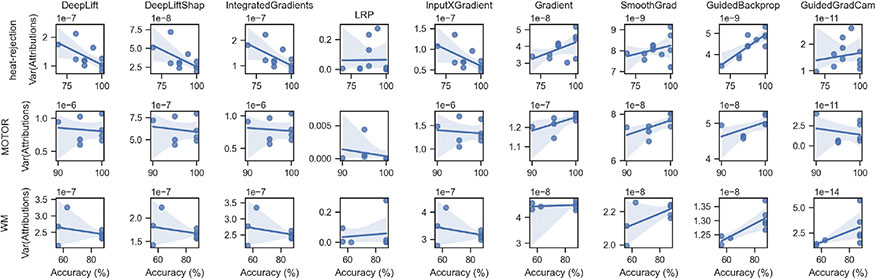
Attribution variance. For each attribution method (columns), we compute the mean variance of its attributions across the 10 trained instances of each dataset’s (rows) model configuration. In general, attributions vary very little across the 10 model instances. We find no consistent association between attribution variance and the models’ test decoding accuracies. Scatter points indicate model means. Lines with indicate linear regression fits with 95% confidence intervals surrounding them.

To ensure that these findings are not specific to our similarity measure, we also computed the Pearson correlation of attribution and BOLD maps and found the same pattern of similarities as well as a strong positive association of the two similarity metrics (Appendix Fig. B.2). We also found that attributions in general vary little across the 10 analyzed model instances (Appendix Figure B.3).

### 2.3. Explanations of reference-based attributions and backward decompositions are more faithful

Next, we were interested in understanding how well the attribution methods perform at capturing the decision process of the trained models. To this end, we analyzed their explanation faithfulness (for details, see Section 1.7.2) by repeatedly masking those voxels of the input that received high relevance values by an attribution method and measuring the rate of occlusion (describing the fraction of total brain voxels whose values are are occluded) at which the decoding accuracy of the trained models drops to chance level (indicating that all information has been removed from the data that the models rely on to accurately decode the mental states).

**Fig. 5.**
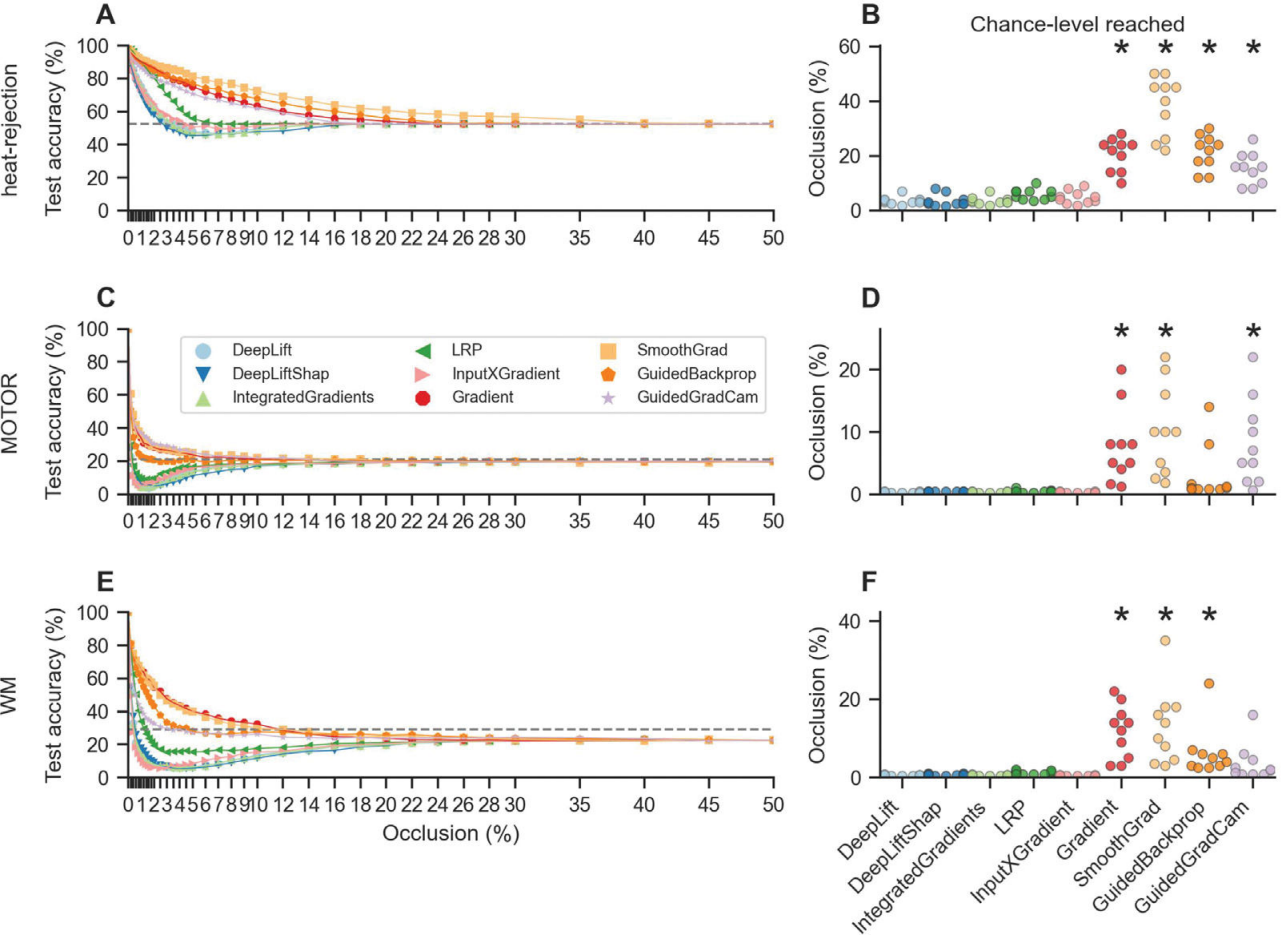
Explanation faithfulness. We estimate the explanation faithfulness of an attribution method by testing whether its attributions correctly identify those voxels of the input that the models rely on in their mental state decoding decisions. To this end, we repeatedly evaluate model test decoding accuracies when occluding different fractions of the input voxel values based on the relevance assigned to them by an attribution method (0% occlusion indicates that no voxel values were occluded while 50% indicates that the values of all input voxels were occluded that received the highest 50% of relevance values). For each attribution method and trained model, we record the occlusion rate at which the models’ test decoding accuracy first drops to chance-level (dashed horizontal lines), indicating that all information has been removed from the data that the model associates with the mental states. A,C,E: DeepLift, DeepLift SHAP, Integrated Gradients, LRP, and InputXGradient exhibit higher explanation faithfulness than Gradient Analysis, SmoothGrad, Guided Backpropagation, and Guided GradCam, as test decoding accuracies decrease more rapidly with increasing occlusion rates for these methods. B,D,F: Accordingly, test decoding accuracies also drop to chance-level at lower occlusion rates for these attribution methods. Colored lines indicate mean test decoding accuracies over the 10 model training runs. Scatter points indicate individual model training runs. Black stars indicate that the distribution of occlusion rates is meaningfully different from the distribution of the DeepLift method (for details, see Section 1.7.4). Colors indicate attribution methods.

This analysis revealed that reference-based attributions and backward decompositions, namely, DeepLift, DeepLift SHAP, Integrated Gradients, LRP, and InputXGradient, generally exhibit higher explanation faithfulness than the considered sensitivity analyses, as the models’ decoding decisions dropped to chance level at lower occlusion rates for these attribution methods than for those of the others (Fig. 5 A–F).

Note that this kind of occlusion can bias models towards consistently misclassifying brain samples as belonging to the “wrong” mental state, as the occluded activity patterns might consistently look to the model like another mental state than the one they actually belong to. Accordingly, model decoding accuracies for the occluded data can drop below chance level (as observed in Fig. 5 E).

### 2.4. Sanity checks for attribution methods

**Fig. 6.**
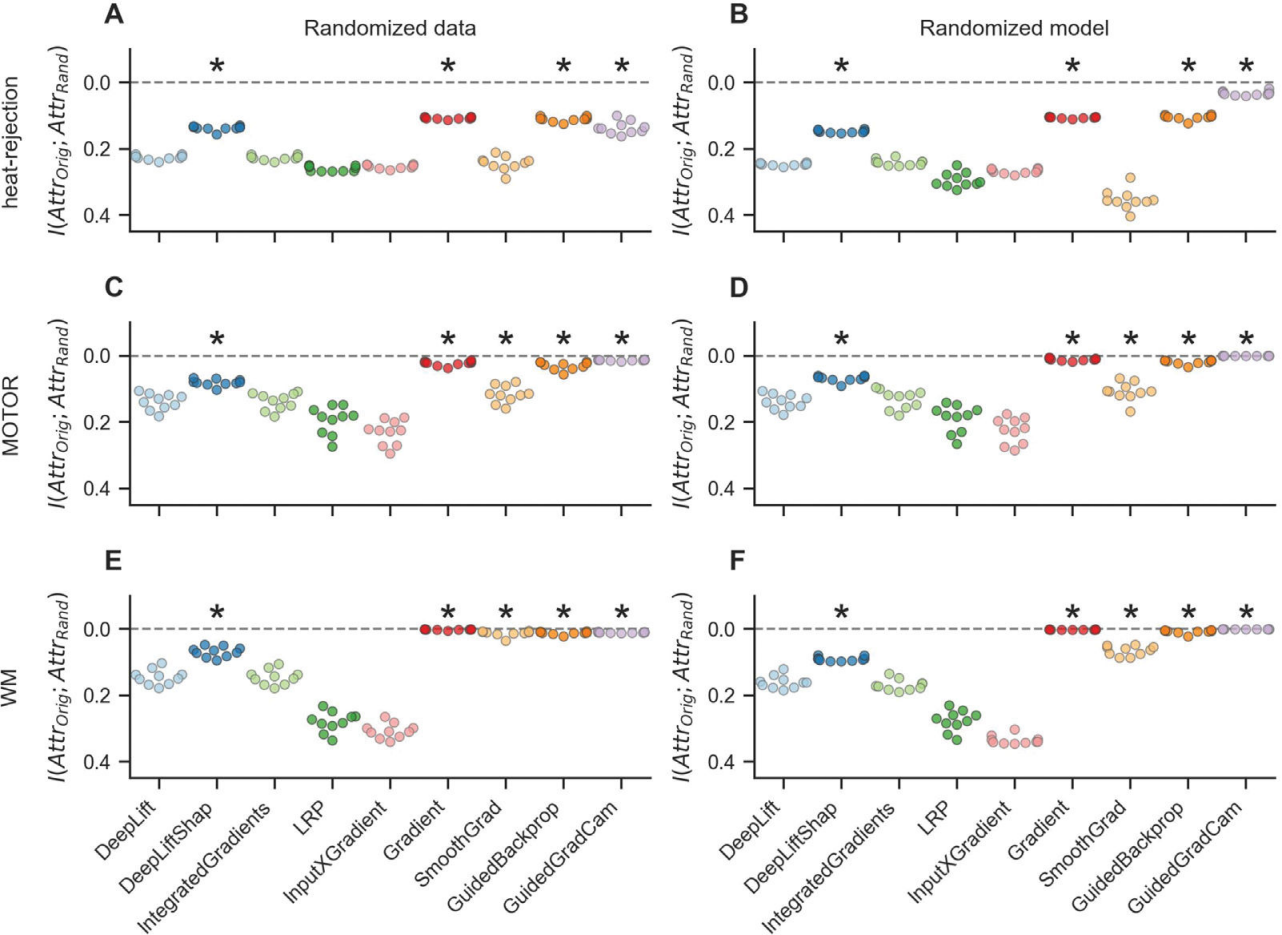
Sanity checks.We perform two sanity for each attribution method by testing whether its attributions are in fact sensitive to the characteristics of the analyzed data and model. Specifically, we compare the attributions of an attribution method for the original model and data to its attributions for a model variant trained on a version of the training data with randomized labels (randomized data; A, C, E) and for a model variant with randomized weights (randomized model; B, D, F). If the attributions of an attribution method are sensitive to the characteristics of the data and model, its attributions for the original model should be different from its attributions for the model trained on randomized data as well as for the model with random weights (resulting in low mutual information scores). Overall, Gradient Analysis, Guided Backpropagation, Guided GradCam, and DeepLift SHAP performed consistently better than the other attribution methods in both sanity checks. Scatter points indicate mean mutual information for each model training run. Gray horizontal lines indicate 0 mutual information. Note that y-axes are inverted. Black stars indicate that a method’s mean mutual information scores are meaningfully smaller than those of DeepLift (for analysis details, see Section 1.7.4). Colors indicate attribution methods.

**Fig. B.4.**
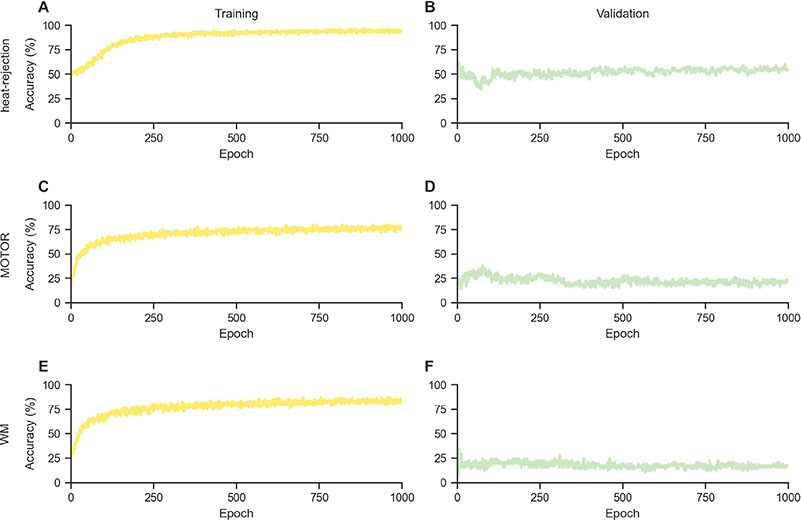
Model performances in the data randomization test. For each dataset, we trained a variant of its model configuration (see Table 1 of the main text) on a variant of the dataset with permuted mental state labels. A,C,E: The trained models learn to accurately decode the randomized mental state labels of the training data. B,D,F: Yet, the models perform close to chance in decoding the randomized labels from the validation data, indicating that they have learned to memorize the mapping between trial-level BOLD maps and mental state labels of the training data. As for our other analyses (see Section 2.1 of the main text), we randomly separated each dataset’s training data into distinct training and validation datasets, comprising 90% and 10% of the full training data respectively. Colors indicate training (yellow) and validation (green) data. Lines indicate decoding accuracies over the course of training. (For interpretation of the references to colour in this figure legend, the reader is referred to the web version of this article.)

Last, we performed two sanity checks for the explanations of the attribution methods to test whether these are in fact sensitive to the characteristics of the analyzed data and model (for details, see Section 1.7.3). In line with the data randomization test, which tests the sensitivity of an explanation towards the characteristics of the analyzed data, we first trained a variant of each datasets’ model configuration on a version of its original training data with randomly shuffled mental state labels. Note that we deliberately used a fixed and very long training period of 1,000 epochs for these model fits because we want the models to overfit their training data by memorizing the assignment of the randomly shuffled labels to the training samples. All models were able to correctly memorize the randomly shuffled mental state labels of their training data, achieving training decoding accuracies of 94.9%, 76.0%, and 84.0% for the heat-rejection, MOTOR, and WM datasets respectively (Appendix Fig. B.4). Importantly, the models’ validation decoding accuracies were still close to chance (55.3%, 22.5%, and 17.5% for the heat-rejection, MOTOR, and WM datasets respectively; Appendix Fig. B.4), showing that the models learned to memorize the random association between labels and training data. We then compared the attributions of each attribution method for the test mental state decoding decisions of these models to their respective attributions for the models trained on the original (not randomized) training data. This comparison showed that the attributions of Gradient Analysis, Guided Backpropagation, Guided GradCam, and DeepLift SHAP were consistently more dissimilar between the two models than those of the other attribution methods, indicating stronger dependence of their attributions on the characteristics of the training data (Fig. 6 A,C,E).

Similarly, in line with the model parameter randomization test, which tests the sensitivity of an explanation towards the characteristics of the analyzed model, we also attributed the test mental state decoding decisions of a randomly initialized variant of each datasets’ model configuration. As for the data randomization test, we compared the resulting attributions of each attribution method to their attributions for the original (not randomized) models. Again, we found that the attributions of Gradient analysis, Guided Backpropagation, Guided GradCam, and DeepLift SHAP showed consistently stronger dependence on the characteristics of the analyzed models, when compared to the other methods, as their attributions for the randomized and not randomized models were generally more dissimilar (Fig. 6 B,D,F).

## 3. Discussion

With this work, we provide insights into the relative performance of prominent types of explanation methods, namely, sensitivity analyses, backward decompositions, and reference-based attributions (see Section 1.6), in mental state decoding analyses with DL models.

To benchmark explanation performances, we use a diverse set of criteria (for an overview, see Table 2): First, we evaluate how well the explanations align with other empirical evidence on the association of the decoded mental states and brain activity by comparing them to the results of a standard GLM analysis of the BOLD data and to the results of a meta-analysis. We find that sensitivity analyses, such Guided Backpropagation, Guided GradCam, Gradient Analysis, and SmoothGrad, provide explanations that are more similar to the results of the GLM/meta-analysis than the explanations of backward decompositions and reference-based attributions. Second, we compare the faithfulness of the explanations by testing whether they identify those voxels of the input that the models rely on to accurately decode the mental states. We find that reference-based attributions and backward decompositions, such as DeepLift, DeepLift SHAP, Integrated Gradients, and LRP, provide explanations that are more faithful than those of the sensitivity analyses. Last, to ensure that the explanations are in fact sensitive to the analyzed model and data, we perform two sanity checks for attribution methods (as suggested by Adebayo et al., 2018) and find that the explanations of Gradient Analysis, Guided Backpropagation, Guided GradCam, and DeepLift SHAP are consistently more sensitive to the characteristics of the analyzed model and data than those of the other explanation methods.

### 3.1. Explanation faithfulness vs. alignment with other empirical evidence

Our findings indicate a gradient between two key characteristics for the explanation of mental state decoding decisions: methods that provide highly faithful explanations, by capturing the model’s decision process well, also provide explanations that align less well with other empirical evidence on the association of the decoded mental states and brain activity. Specifically, our results indicate that highly faithful explanations do not necessarily identify all voxels of the brain whose activity patterns are in fact associated with the decoded mental states. To make sense of this finding, it is important to remember that functional neuroimaging data exhibit strong spatial correlations, such that individual mental states are often associated with the activity of large clusters of voxels. DL models trained to identify these mental states from neuroimaging data will likely view some of this activity as redundant, as the activity of a subset of those voxels suffices to correctly identify the mental states. In these situations, any explanation with perfect faithfulness will not identify all voxels of the input whose activity is in fact associated with the decoded mental state but solely the subset of voxels whose activity the model used as evidence for its decoding decision. Accordingly, attribution methods with high explanation faithfulness, such as reference-based attributions and backward decompositions, do not necessarily produce explanations that align well with the results of other analysis approaches for BOLD data, which seek to identify all voxels whose activity is associated with the mental states. By contrast, we found that sensitivity analyses, such as Guided GradCam, Guided Backpropagation, Gradient Analysis, and SmoothGrad, provide explanations that are less faithful but more in line with the results of a GLM/meta-analysis. Sensitivity analyses are less concerned with identifying the specific contribution of each input voxel to a decoding decision and instead focus on identifying how sensitively a model’s decision responds to (i.e., changes with) the activity of each voxel. Consequently, sensitivity analyses identify a broader set of voxels whose activity the model takes into account when decoding the mental state, resulting in explanations that align better with the results of analysis approaches that seek to identify all voxels whose activity pattern is associated with the mental state.

### 3.2. Recommendations for explanation methods in mental state decoding

Based on these findings, we make a twofold recommendation for the application of explanation methods in mental state decoding:

If the focus of an explanation analysis is on understanding the decision process of the decoding model by identifying the parts of the input that are most relevant for the model’s decision, we recommend the application of reference-based attributions. In particular, we recommend DeepLift SHAP because its explanations were highly faithful in our analyses while also performing well in the two sanity checks. As DeepLift SHAP can be computationally expensive for larger datasets, because it requires comparing each data sample to many baselines (see Section 1.6), we additionally recommend the application of standard DeepLift in situations where computational costs are of concern. We further recommend the application of Integrated Gradients for situations in which DeepLift is difficult to implement (e.g., the Deeplift implementation provided by Captum (Kokhlikyan et al., 2020) currently only supports a limited number on non-linear activation functions), as Integrated Gradients can be applied to any differentiable model and its explanations have been shown to be theoretically and empirically closely related to those of DeepLift (Ancona et al., 2018) (but are also more more expensive to compute).

By contrast, if the goal of an explanation analysis is to understand the association between the BOLD data and studied mental states, and DL models are merely used as a tool to study this association, we recommend the application of sensitivity analyses because their explanations align best with other empirical evidence on the association between the studied mental states and brain activity. Particularly, for CNN models, which primarily use *ReLU* activation functions, we recommend Guided Backpropagation because its explanations exhibit the overall highest similarity to the results of our GLM/meta-analysis while also performing well in the two sanity checks. For other model architectures without *ReLU* activation functions, we recommend standard Gradient Analysis, as its explanations also have comparably high similarity to the GLM/meta-analysis results and perform well in the two sanity checks.

### 3.3. Caution in the explanation of complex models

Last, we would like to advocate for caution in any explanation of the mental state decoding decisions of DL models. DL models have an unmatched ability to learn from and represent complex data. Consequently, their learned mappings between input data and mental states can be highly complex and counterintuitive. For example, recent empirical work has shown that DL models trained in mental state decoding analyses can identify individual mental states through voxels that exhibit meaningfully stronger activity in these states as well as voxels with meaningfully reduced activity in these states (Thomas et al., 2021). Accordingly, an explanation of their decoding decisions leads to explanations that are inconsistent with the results of a standard GLM contrast analysis of the same BOLD by assigning high relevance to voxels that receive both positive and negative weights by the GLM analysis. To understand how a model’s weighting of the input in its decoding decision relates to the characteristics of the input data, it is therefore essential to compare the explanations of any explanation method to the results of standard analyses of the BOLD data (e.g., with linear models; Friston et al., 1994; Grosenick et al., 2013; Kriegeskorte et al., 2006) as well as to related empirical findings (e.g., as provided by NeuroQuery and NeuroSynth; Docks et al., 2020; Yarkoni et al., 2011).

Similarly, a wealth of empirical work has demonstrated that DL models are prone to learning simple shortcuts (or confounds) from their training data, which do not generalize to other datasets (for a detailed discussion, see Geirhos et al., 2020). A prominent example is a study that trained DL models to identify pneumonia from chest X-rays (Zech et al., 2018). While the models performed well in the training data, comprising X-rays from few hospitals, their performance meaningfully decreased for X-rays from new hospitals. By applying attribution methods to the classification decisions of the trained models, the authors were able to show that the models learned to accurately identify the hospital system that was used to acquire an X-ray, in combination with the specific department, allowing them to make accurate predictions on aggregate simply by learning the overall prevalence rates of these departments. Similar examples are imaginable in functional neuroimaging, as recently suggested by Chyzhyk et al. (2022) who hypothesize that biomarker models for specific disease conditions could learn to distinguish patients from controls by their generally increased head motion.

For these reasons, we echo a recent call of machine learning researchers to always consider whether the application of complex models (such as DL models) is necessary to answer the research question at hand, or whether the application of simpler models, with better explainability, could suffice (Rudin, 2019). While we do believe that DL models hold a high promise for mental state decoding research, e.g., with their ability to learn from large-scale neuroimaging datasets (Schulz et al., 2022; Thomas et al., 2022b), we also believe that many common types of mental state decoding analysis, which solely focus on few mental states in tens to a hundred of individuals, can be well-addressed with simpler decoding models with better explainability (e.g., Grosenick et al., 2013; Hoyos-Idrobo et al., 2018; Kriegeskorte et al., 2006; Michel et al., 2011; Schulz et al., 2020).

### 3.4. Conclusion

In sum, we hope that with this work we can provide some insights into the relative performance of prominent explanation methods in mental state decoding, and enable neuroimaging researchers to make an informed choice among existing explanation methods in situations where an explanation of the mental state decoding decision of a DL model is needed.

## Figures and Tables

**Table 1:** Best-performing model and optimization configurations. For each dataset, the number of convolution layers, number of kernels per convolution layer, size of the kernels, training mini-batch size (BS), learning rate (LR), and dropout rate are shown for the configuration with lowest $\lambda_{i}$ (indicating best performance; see Section 1.5.2).

Dataset	#Layers	#Kernels	Kernel size	BS	LR	Dropout

heat-rejection	4	8	5	32	0.0003	0.5
MOTOR	4	4	3	32	0.001	0.25
WM	3	4	5	32	0.001	0.5

**Table 2:** Results overview. “✓” indicates that the explanations of an attribution method performed comparably well in the respective evaluation, as determined by our statistical comparison (see Section 1.7.4), while “✗” indicates that they did not.

Evaluation	DeepLift	DeepLift SHAP	Integr. Gradients	LRP	InputXGradient	Gradient	SmoothGrad	Guided Backprop.	Guided GradCam

Aligned w/ other evidence?	✗	✗	✗	✗	✗	✓	✓	✓	✓
Faithful?	✓	✓	✓	✓	✓	✗	✗	✗	✗
Sensitive to data?	✗	✓	✗	✗	✗	✓	✗	✓	✓
Sensitive to model?	✗	✓	✗	✗	✗	✓	✗	✓	✓
*N*(✓)	1	3	1	1	1	3	1	3	3

## Data Availability

I have shared the link to my data/code in the manuscript.

## Footnotes

1
https://db.humanconnectome.org/data/projects/HCP1200

2
https://github.com/cocoanlab/interpret_ml_neuroimaging

## Appendix A. Methods

### A1. Fmriprep details

Results included in this manuscript come from preprocessing performed using *fMRIPrep* 20.2.3 (Esteban et al. (2019); Esteban et al. (2018); RRID:SCR_016216), which is based on *Nipype* 1.6.1 (Gorgolewski et al. (2011); Gorgolewski et al. (2018); RRID:SCR_002502).

Anatomical data preprocessing The T1-weighted (T1w) images were corrected for intensity non-uniformity (INU) with N4BiasFieldCorrection (Tustison et al., 2010), distributed with ANTs 2.3.3 (Avants et al., 2008, RRID:SCR_004757), and used as T1w-reference throughout the workflow. The T1w-reference was then skull-stripped with a *Nipype* implementation of the antsBrainExtraction.sh workflow (from ANTs), using OASIS30ANTs as target template. Brain tissue segmentation of cerebrospinal fluid (CSF), white-matter (WM) and gray-matter (GM) was performed on the brain-extracted T1w using fast (FSL 5.0.9, RRID:SCR_002823, Zhang et al., 2001). Volume-based spatial normalization to two standard spaces (MNI152NLin2009cAsym, MNI152NLin6Asym) was performed through nonlinear registration with antsRegistration (ANTs 2.3.3), using brain-extracted versions of both T1w reference and the T1w template. The following templates were selected for spatial normalization: *ICBM 152 Nonlinear Asymmetrical template version 2009c* [Fonov et al. (2009), RRID:SCR_008796; TemplateFlow ID: MNI152NLin2009cAsym], *FSL’s MNI ICBM 152 non-linear 6th Generation Asymmetric Average Brain Stereotaxic Registration Model* [Evans et al. (2012), RRID:SCR_002823; TemplateFlow ID: MNI152NLin6Asym],

Functional data preprocessing For each of the BOLD runs found per subject, the following preprocessing was performed. First, a reference volume and its skull-stripped version were generated using a custom methodology of *fMRIPrep*. Susceptibility distortion correction (SDC) was omitted. The BOLD reference was then co-registered to the T1w reference using flirt (FSL 5.0.9, Jenkinson and Smith, 2001) with the boundary-based registration (Greve and Fischl, 2009) cost-function. Co-registration was configured with nine degrees of freedom to account for distortions remaining in the BOLD reference. Head-motion parameters with respect to the BOLD reference (transformation matrices, and six corresponding rotation and translation parameters) are estimated before any spatiotemporal filtering using mcflirt (FSL 5.0.9, Jenkinson et al., 2002). The BOLD time-series (including slice-timing correction when applied) were resampled onto their original, native space by applying the transforms to correct for head-motion. These resampled BOLD time-series will be referred to as *preprocessed BOLD in original space*, or just *preprocessed BOLD*. The BOLD time-series were resampled into standard space, generating a *preprocessed BOLD run in MNI152NLin2009cAsym space*. First, a reference volume and its skull-stripped version were generated using a custom methodology of *fMRIPrep*. Automatic removal of motion artifacts using independent component analysis (ICA-AROMA, Pruim et al., 2015) was performed on the *preprocessed BOLD on MNI space* time-series after removal of non-steady state volumes and spatial smoothing with an isotropic, Gaussian kernel of 6mm FWHM (full-width half-maximum). Corresponding “non-aggresively” denoised runs were produced after such smoothing. Additionally, the “aggressive” noise-regressors were collected and placed in the corresponding confounds file. Several confounding time-series were calculated based on the *preprocessed BOLD*: framewise displacement (FD), DVARS and three region-wise global signals. FD was computed using two formulations following Power (absolute sum of relative motions, Power et al. (2014)) and Jenkinson (relative root mean square displacement between affines, Jenkinson et al. (2002)). FD and DVARS are calculated for each functional run, both using their implementations in *Nipype* (following the definitions by Power et al., 2014). The three global signals are extracted within the CSF, the WM, and the whole-brain masks. Additionally, a set of physiological regressors were extracted to allow for component-based noise correction (*CompCor*, Behzadi et al., 2007). Principal components are estimated after high-pass filtering the *preprocessed BOLD* time-series (using a discrete cosine filter with 128s cut-off) for the two *CompCor* variants: temporal (tCompCor) and anatomical (aCompCor). tCompCor components are then calculated from the top 2% variable voxels within the brain mask. For aCompCor, three probabilistic masks (CSF, WM and combined CSF+WM) are generated in anatomical space. The implementation differs from that of Behzadi et al. in that instead of eroding the masks by 2 pixels on BOLD space, the aCompCor masks are subtracted a mask of pixels that likely contain a volume fraction of GM. This mask is obtained by thresholding the corresponding partial volume map at 0.05, and it ensures components are not extracted from voxels containing a minimal fraction of GM. Finally, these masks are resampled into BOLD space and binarized by thresholding at 0.99 (as in the original implementation). Components are also calculated separately within the WM and CSF masks. For each CompCor decomposition, the k components with the largest singular values are retained, such that the retained components’ time series are sufficient to explain 50 percent of variance across the nuisance mask (CSF, WM, combined, or temporal). The remaining components are dropped from consideration. The head-motion estimates calculated in the correction step were also placed within the corresponding confounds file. The confound time series derived from head motion estimates and global signals were expanded with the inclusion of temporal derivatives and quadratic terms for each (Satterthwaite et al., 2013). Frames that exceeded a threshold of 0.5 mm FD or 1.5 standardised DVARS were annotated as motion outliers. All resamplings can be performed with *a single interpolation step* by composing all the pertinent transformations (i.e. head-motion transform matrices, susceptibility distortion correction when available, and co-registrations to anatomical and output spaces). Gridded (volumetric) resamplings were performed using antsApplyTransforms (ANTs), configured with Lanczos interpolation to minimize the smoothing effects of other kernels (Lanczos, 1964). Non-gridded (surface) resamplings were performed using mri_vol2surf (FreeSurfer).

Many internal operations of *fMRIPrep* use *Nilearn* 0.6.2 (Abraham et al., 2014, RRID:SCR_001362), mostly within the functional processing workflow. For more details of the pipeline, see the section corresponding to workflows in fMRIPrep’s documentation.

#### Copyright Waiver

The above boilerplate text was automatically generated by fMRIPrep with the express intention that users should copy and paste this text into their manuscripts *unchanged*. It is released under the CC0 license.
